# 

**Published:** 1894-08

**Authors:** 


					﻿NOTIVE
All artic\^p>r pul^aatiap, books Aln^nrieie, bueineen conimunicatitni, ele.,
must be eent to Pdit^r, n^^Locuxt Mntéf, Philadelphia.
Subtcribert willf^ute notiee^Utajathix Jmtrnal will be eent until arreare
are paid and it	diet^^intued.
				

